# Predictors of 30-Day Healthcare Utilization After Telemedicine Management of Minimal Traumatic Intracranial Hemorrhage

**DOI:** 10.7759/cureus.112591

**Published:** 2026-07-13

**Authors:** Zac W Riggenbach, Jennifer M Teng, Abigail Kelly, Brooklyn Williams, Jonathan Lutgens, Oladapo Akinmoladun, Paul Inouye

**Affiliations:** 1 General Surgery, Madigan Army Medical Center, Tacoma, USA; 2 Pediatrics, Madigan Army Medical Center, Tacoma, USA; 3 General Surgery, St. Joseph Medical Center, Tacoma, USA

**Keywords:** intracranial hemorrhage (ich), mild traumatic brain injury (mtbi), patient readmission, telemedicine, trauma aftercare, trauma follow-up

## Abstract

Introduction

Telemedicine protocols for minimal traumatic intracranial hemorrhage (ICH) enable peripheral management without transfer to trauma centers, but post-discharge utilization patterns among these patients remain poorly characterized. This study evaluated factors associated with 30-day readmission and unplanned re-encounter among adult patients managed after implementation of a telemedicine minimal head bleed protocol.

Methods

A retrospective cohort study was conducted of adult patients with minimal traumatic ICH managed without transfer under a telemedicine protocol at a regional trauma system between July 2021 and December 2023. The primary outcome was 30-day readmission. The secondary outcome was any 30-day unplanned re-encounter. Follow-up was classified as primary care, trauma clinic, surgical subspecialty, or non-surgical subspecialty. Unadjusted comparisons used Fisher's exact testing and Mann-Whitney U testing. Exploratory logistic regression models adjusted for age, sex, and injury severity score (ISS) were performed for each follow-up category. Firth's penalized logistic regression was used as a sensitivity analysis given the limited number of readmission events.

Results

Among 241 adult patients, 24 (10.0%) experienced a 30-day readmission, and 61 (25.3%) experienced an unplanned re-encounter. Age, sex, and ISS were not significantly associated with either outcome. Any subspecialty follow-up was associated with increased odds of readmission (adjusted odds ratio (OR), 2.73; 95% CI, 1.10-6.77; p = 0.031), confirmed by Firth's penalized regression (OR, 2.72; 95% CI, 1.09-6.55; p = 0.033). Non-surgical subspecialty follow-up demonstrated the strongest association (adjusted OR, 7.15; 95% CI, 1.80-28.50; p = 0.005), while surgical subspecialty follow-up was not significantly associated with readmission (adjusted OR, 1.39; 95% CI, 0.47-4.08; p = 0.551). No measured variable was significantly associated with unplanned re-encounter. Re-encounters were clinically heterogeneous, including recurrent falls and neurologic symptoms.

Conclusions

Among patients managed via a telemedicine minimal head bleed protocol, age, sex, and ISS were not significantly associated with post-discharge healthcare utilization. Subspecialty follow-up, particularly non-surgical subspecialty care, may serve as a practical marker of post-discharge clinical complexity and could help identify patients for future evaluation of targeted transitional-care strategies. These early findings warrant further studies and validation.

## Introduction

Traumatic injury has long been conceptualized as an acute, time-limited event; however, modern trauma epidemiology increasingly supports its characterization as a chronic, relapsing condition marked by recurrent morbidity, repeat injury, and prolonged psychosocial impact [1-3]. Viewing trauma through this lens shifts the focus of trauma systems from single-episode stabilization toward longitudinal recovery, reinjury prevention, and social reintegration. Long-term studies demonstrate that individuals hospitalized after trauma have up to a 44% likelihood of sustaining another injury within several years, a phenomenon termed “trauma recidivism” [2]. This pattern is driven by persistent behavioral and social risk factors, such as alcohol use, unstable housing, and limited access to preventive care, which mirror the self-perpetuating mechanisms observed in chronic disease [1-3]. Readmissions and recurrent emergency department (ED) visits represent measurable manifestations of this chronic course. Smith et al. reported 30-day readmission rates of 11% among trauma inpatients, and ED revisit rates of 13-14%, most often driven by wound complications, musculoskeletal complaints, psychiatric distress, inadequate pain control, or gaps in social support [4-7].

Unplanned trauma readmissions are costly and frequently preventable. The average per-readmission cost exceeds $10,000, with national expenditures surpassing $313 million annually [8]. Neiman et al. estimated that roughly one in five trauma readmissions could be avoided, underscoring the need for more deliberate outpatient continuity strategies [8]. Inpatient data clearly demonstrate the value of trauma-system organization. Staudenmayer et al. found that care in designated trauma centers independently predicted lower unplanned readmission compared with care at non-trauma hospitals, emphasizing that trauma-specific infrastructure improves outcomes even beyond the acute admission [9]. Whether these benefits extend into the post-discharge phase, however, remains uncertain.

Outpatient follow-up has also served as a quality metric, yet emerging evidence suggests that who provides follow-up matters as much as if it occurs. Smith et al. studied 2,266 trauma patients and found no significant reduction in 30-day ED utilization or readmissions among those who obtained generic outpatient follow-up [4]. The authors suggested that non-trauma providers may be ill-equipped to address the complex clinical and psychosocial needs of trauma survivors. In contrast, structured trauma-directed programs demonstrate tangible benefit. Hall et al. implemented a trauma transitional care program incorporating inpatient identification, early phone outreach, trauma clinic scheduling, and social needs screening, resulting in a significant reduction in 30-day unplanned readmissions [10]. Among patients with minimal traumatic intracranial hemorrhage, post-discharge healthcare utilization is likely multifactorial. While delayed neurologic complications remain an important consideration, recurrent healthcare encounters may also reflect persistent post-concussive symptoms, medical comorbidities, recurrent falls, functional limitations, or psychosocial vulnerabilities. Distinguishing these contributors is important when evaluating healthcare utilization after telemedicine-guided management.

Telemedicine may present a new confounder to the current research surrounding trauma recidivism and follow-up care. Telemedicine has become increasingly important to trauma medical care over the past decade and has accelerated during and after the COVID-19 pandemic [11]. While telemedicine has demonstrated benefits during the acute management of trauma patients, comparatively little is known about post-discharge healthcare utilization or trauma recidivism among patients managed through telemedicine pathways [12,13].

The primary objective of this study was to characterize 30-day post-discharge healthcare utilization among adults with minimal traumatic intracranial hemorrhage managed without transfer through a regional telemedicine minimal head bleed protocol, using 30-day hospital readmission as the primary outcome. The secondary objective was to characterize 30-day unplanned healthcare re-encounters. As exploratory analyses, we evaluated demographic, injury-related, and outpatient follow-up characteristics associated with each outcome.

## Materials and methods

Study design and setting

The telemedicine protocol employed in this study was developed to reduce unnecessary patient transfers to a centrally located Level II trauma center by facilitating remote management of select patients with minimal traumatic intracranial hemorrhage. This regional trauma system consists of a central Level II trauma center serving as the referral center for several outlying community hospitals. Management decisions were guided by a standardized institutional minimal head bleed protocol, which was used collaboratively by the trauma surgeon at the receiving trauma center and the treating provider at the referring facility to determine whether transfer was indicated. Consultation consisted of a real-time physician-to-physician telephone discussion supplemented by an asynchronous review of the patient’s electronic medical record, clinical documentation, laboratory results, and neuroimaging. Patients meeting protocol criteria for local management remained at the referring facility with ongoing availability for additional consultation as needed. The protocol was designed for patients with small traumatic intracranial hemorrhages not requiring transfer for neurosurgical evaluation, including select traumatic subarachnoid hemorrhages, subdural hematomas, epidural hematomas, intraparenchymal hemorrhages, and cerebral contusions without significant mass effect or other indications for operative neurosurgical intervention. Follow-up type and timing were determined collaboratively by the trauma team at the central Level II trauma center and clinicians at the referring facility.

Following institutional IRB approval, we conducted a retrospective cohort study of patients evaluated under this protocol between July 1, 2021, and December 2023. Patients were identified through the trauma registry, with eligibility determined based on initial presentation to an outlying facility, no transfer to the Level II trauma center or other tertiary care facility, and documentation of a trauma team consultation. Only cases classified as minimal head bleeds or skull fractures, defined by including patients with either ICD 10 codes S06.0-S06.9 or S02.0-S02.19, were included [14]. Exclusion criteria comprised age under 18, lack of a unique trauma identifier, transfer to the trauma center, or direct presentation to the trauma center. No missing data were present for variables included in the primary analyses.

Outcomes

The primary outcome was 30-day readmission, defined as any inpatient readmission within 30 days of the index encounter and analyzed as a binary outcome (any readmission vs. none). Readmission was selected as the primary outcome because it represents a higher-acuity, more resource-intensive event with greater clinical and policy relevance than emergency department revisits alone. The secondary outcome was any 30-day unplanned re-encounter, defined as an unscheduled emergency department, urgent care, or other unplanned healthcare encounter within 30 days of the index encounter. Although unplanned encounters were also available as a count variable, the primary re-encounter analysis used a binary outcome to reflect whether the patient returned for any unplanned care. Planned outpatient encounters were analyzed separately and included scheduled follow-up visits with primary care, specialty clinics, trauma clinic, or other outpatient services. Planned encounters were not treated as adverse outcomes but rather as markers of post-discharge care complexity.

Follow-up and re-encounter classification

Follow-up data were collected by reviewing the hospital system's electronic healthcare record. Planned follow-up encounters were grouped into five non-mutually exclusive categories: primary care, trauma clinic, surgical subspecialty, non-surgical subspecialty, and any subspecialty follow-up as outlined in Table 1. Because patients could have more than one type of follow-up, detailed follow-up categories were not mutually exclusive.

**Table 1:** Follow-up categories This table describes the five categorizations of follow-up that patients managed via the telemedical protocol received and their relative frequency.

Category Title	Description
Primary Care	Family Medicine, Internal Medicine, General Practice
Trauma Follow-up	Designated Trauma Clinic, Trauma Surgery, or General Surgery
Surgical Subspecialty	Neurosurgery, Orthopedics, Plastics, ENT/Oral and Maxillofacial Surgery
Non-surgical Subspecialty	Neurology, Cardiology, Pulmonology, Rehabilitation and Physical Medicine, Occupational or Physical Therapy
Any Subspecialty Follow-Up	Either Surgical or Non-surgical Subspecialty Follow-Up

Similarly, unplanned re-encounters were further classified into appropriate categories for further analysis. The categories included repeat fall, trauma, or pain-related complaint; medical non-neurologic complaint; headache or neurologic symptoms; recurrent bleed or imaging concern; psychiatric, social, or palliative presentation; and other or unclear. This categorization was exploratory and intended to describe the heterogeneity of return visits.

Statistical analysis

Continuous variables were summarized using medians with interquartile ranges (IQR) and compared using the Mann-Whitney U test. Categorical variables were summarized as counts and percentages and compared using Fisher's exact test, which was selected because several subgroups were small. Unadjusted analyses compared age, sex, ISS, and follow-up categories by readmission and unplanned re-encounter status. An exploratory logistic regression was then performed to assess whether the follow-up category remained associated with outcomes after adjustment for baseline demographic and injury-related variables. Each model included the follow-up exposure of interest, age (modeled per decade), sex, and ISS (modeled per 5-point increase) to improve the interpretability of continuous covariates.

To assess the stability of findings in the setting of low event counts, Firth's penalized logistic regression was performed as a sensitivity analysis for the readmission outcome. Firth's models included the same four covariates as the primary models. Each Firth model included 24 readmission events and 4 covariates, corresponding to an events-per-variable ratio (EPV) of 6. Profile penalized-likelihood confidence intervals were used for the Firth estimates.

All analyses were performed using Python version 3.13.13 (https://www.python.org/). Data cleaning and management were performed using pandas version 2.2.3 and NumPy version 2.3.5. Statistical testing and model estimation were performed using SciPy version 1.17.1. Standard logistic regression models were fit using maximum likelihood estimation and Firth's penalized logistic regression. Statistical significance was defined as p < 0.05. Analyses were conducted with the assistance of generative AI and AI-assisted analytic tools, including ChatGPT (OpenAI; San Francisco, CA, USA) and Julius AI (San Francisco, CA, USA), for code generation, statistical workflow development, and manuscript editing. All code, analytic decisions, model outputs, figures, tables, and written content were reviewed and verified by the study investigators. 

## Results

Cohort characteristics

A total of 246 patients were managed after protocol implementation. A total of 5 patients less than 18 years of age were excluded from the dataset. After excluding 5 patients younger than 18 years, the final analytic cohort included 241 adult patients. The cohort had a median age of 62 years (IQR 38-77) and a median ISS of 5 (IQR 1-10), consistent with a low-acuity, minimal head bleed population. Male patients represented 57.3% (138/241) of the dataset. Overall, 24 patients (10.0%; 95% CI: 6.5-14.5%) experienced a 30-day readmission, and 61 patients (25.3%; 95% CI: 20.0-31.2%) experienced at least one unplanned 30-day re-encounter. 

The overall follow-up rate was 38.6% (93/241). Primary care follow-up occurred in 23.7% (57/241), followed by any subspecialty follow-up at 19.9% (48/241), of whom 16.2% (39/241) had surgical subspecialty follow-up, and 4.1% (10/241) had non-surgical subspecialty follow-up. Trauma clinic follow-up was uncommon, occurring in 9 patients (3.7%), as shown in Table 2.

**Table 2:** Baseline characteristics by 30-day readmission status Continuous variables were compared using Mann-Whitney U testing. Categorical variables were compared using Fisher's exact testing; Fisher's exact odds ratios are reported as the test statistic. Because follow-up categories were not mutually exclusive, counts across follow-up categories are not additive.

Variable	Readmitted (n=24)	Not readmitted (n=217)	Test statistic	p-value
Age, median (IQR)	53.5 (25-82.2)	62 (39-76)	U=2400.5	0.531
ISS, median (IQR)	5 (1-10)	5 (1-10)	U=2656.5	0.871
Male sex, n (%)	15 (62.5%)	123 (56.7%)	Fisher OR=1.274	0.667
Any follow-up, n (%)	14 (58.3%)	78 (35.9%)	Fisher OR=2.495	0.045*
Primary care follow-up, n (%)	7 (29.2%)	50 (23.0%)	Fisher OR=1.375	0.612
Any subspecialty follow-up, n (%)	9 (37.5%)	39 (18.0%)	Fisher OR=2.738	0.031*
Surgical subspecialty follow-up, n (%)	5 (20.8%)	34 (15.7%)	Fisher OR=1.416	0.558
Non-surgical subspecialty follow-up, n (%)	4 (16.7%)	6 (2.8%)	Fisher OR=7.033	0.011*
Trauma clinic follow-up, n (%)	0 (0.0%)	9 (4.1%)	Fisher OR=0.0	0.605

Age, sex, and ISS were not significantly associated with 30-day readmission. Patients who were readmitted had a median age of 53.5 years (IQR, 25.0-82.3), compared with 62.0 years (IQR, 39.0-76.0) among those not readmitted (p = 0.531). Median ISS was 5 (IQR, 1-10) in both groups (p = 0.871). Male sex was present in 15 of 24 readmitted patients (62.5%) and 123 of 217 non-readmitted patients (56.7%; p = 0.667). Age, sex, and ISS were also not significantly associated with unplanned 30-day re-encounter. Patients with an unplanned re-encounter had a median age of 60.0 years (IQR, 40.0-78.0), compared with 62.0 years (IQR, 37.0-76.0) among those without (p = 0.797). Median ISS was numerically higher among patients with a re-encounter (8 (IQR, 1-10) vs. 5 (IQR, 1-10)), though this difference was not statistically significant (p = 0.292). Males composed 60.7% of re-encounters (37/61) and 56.1% (101/180) of those without re-encounters (p=0.553). 

Thirty-day readmission

Readmission was more common among patients with any subspecialty follow-up: 9 (18.8%) versus 15 (7.8%) among those without subspecialty follow-up (p = 0.031). In an exploratory logistic regression model adjusted for age, sex, and ISS, any subspecialty follow-up remained associated with higher odds of readmission (adjusted OR, 2.73; 95% CI, 1.10-6.77; p = 0.031). Age, sex, and ISS were not significantly associated with readmission in the Firth penalized logistic regression model (OR, 2.72; 95% CI, 1.09-6.55; p = 0.033).

When subspecialty follow-up was subdivided, the strongest association was observed among patients with non-surgical subspecialty follow-up. Readmission occurred in 4 of 10 patients (40.0%) with non-surgical subspecialty follow-up, compared with 20 (8.7%) without (p = 0.011). After adjustment for age, sex, and ISS, non-surgical subspecialty follow-up was associated with an adjusted OR of 7.15 (95% CI, 1.80-28.50; p = 0.005). The wide confidence intervals reflect the small size of the non-surgical subspecialty subgroup (n = 10) and should be interpreted accordingly.

Surgical subspecialty follow-up was not significantly associated with readmission. Readmission occurred in 5 patients (12.8%) with surgical subspecialty follow-up, compared with 19 (9.4%) without (p = 0.558). In the adjusted model, surgical subspecialty follow-up was not associated with readmission (adjusted OR, 1.39; 95% CI, 0.47-4.08; p = 0.551) as highlighted in Table 3. Trauma clinic follow-up was uncommon (n = 9), and no readmissions occurred among these patients. The small sample size precludes meaningful statistical inference regarding trauma clinic follow-up and readmission.

**Table 3:** Cohort readmission characteristics Adjusted and Firth's penalized models were fit separately for each follow-up exposure variable and adjusted for age (per decade), sex, and ISS (per 5-point increase). Because follow-up categories were not mutually exclusive, patients could contribute to more than one exposure-specific model.

Follow-up exposure	Readmission rate exposed vs unexposed	Unadjusted OR (95% CI)	Unadjusted test statistic	Unadjusted p-value	Firth's penalized OR (95% CI)	Firth's test statistic	Firth's p-value
Any subspecialty follow-up	9/48 (18.8%) vs 15/193 (7.8%)	2.74 (1.13-6.61)	Wald z = 2.24	0.025	2.72 (1.09-6.55)	4.53	0.033*
Non-surgical subspecialty follow-up	4/10 (40.0%) vs 20/231 (8.7%)	7.03 (1.82-27.11)	Wald z = 2.83	0.005	6.98 (1.79-25.34)	7.31	0.007*
Surgical subspecialty follow-up	5/39 (12.8%) vs 19/202 (9.4%)	1.42 (0.5-4.02)	Wald z = 0.65	0.513	1.47 (0.48-3.88)	0.51	0.475

Thirty-day unplanned re-encounter

**Figure 1:**
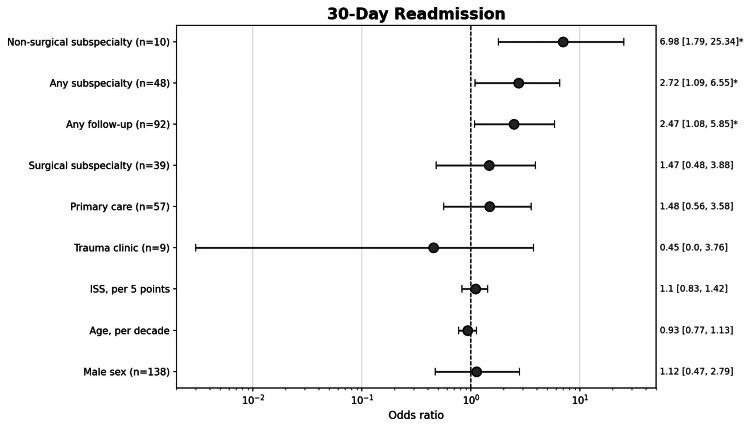
Risk factors for 30-day readmission Each follow-up exposure was analyzed in a separate Firth's penalized logistic regression model adjusted for age, sex, and ISS [15]. Follow-up categories were not mutually exclusive; therefore, patients could contribute to multiple exposure-specific models, but no model simultaneously included multiple follow-up categories. ISS: injury severity score

Unplanned re-encounters were not significantly associated with any measured demographic, injury-related, or follow-up variable. Patients with any subspecialty follow-up had a re-encounter rate of 14 of 48 (29.2%), compared with 47 of 193 (24.4%) without (p = 0.578). In the adjusted model, any subspecialty follow-up was not associated with re-encounter (adjusted OR, 1.21; 95% CI, 0.58-2.52; p = 0.606). Surgical subspecialty follow-up (adjusted OR, 0.94; 95% CI, 0.41-2.14; p = 0.881) and non-surgical subspecialty follow-up (adjusted OR, 2.18; 95% CI, 0.59-7.99; p = 0.241) were also not significantly associated with re-encounter. Age, sex, and ISS were not associated with re-encounter in any model, as shown in Figure 1.

Reasons for unplanned re-encounter

**Figure 2:**
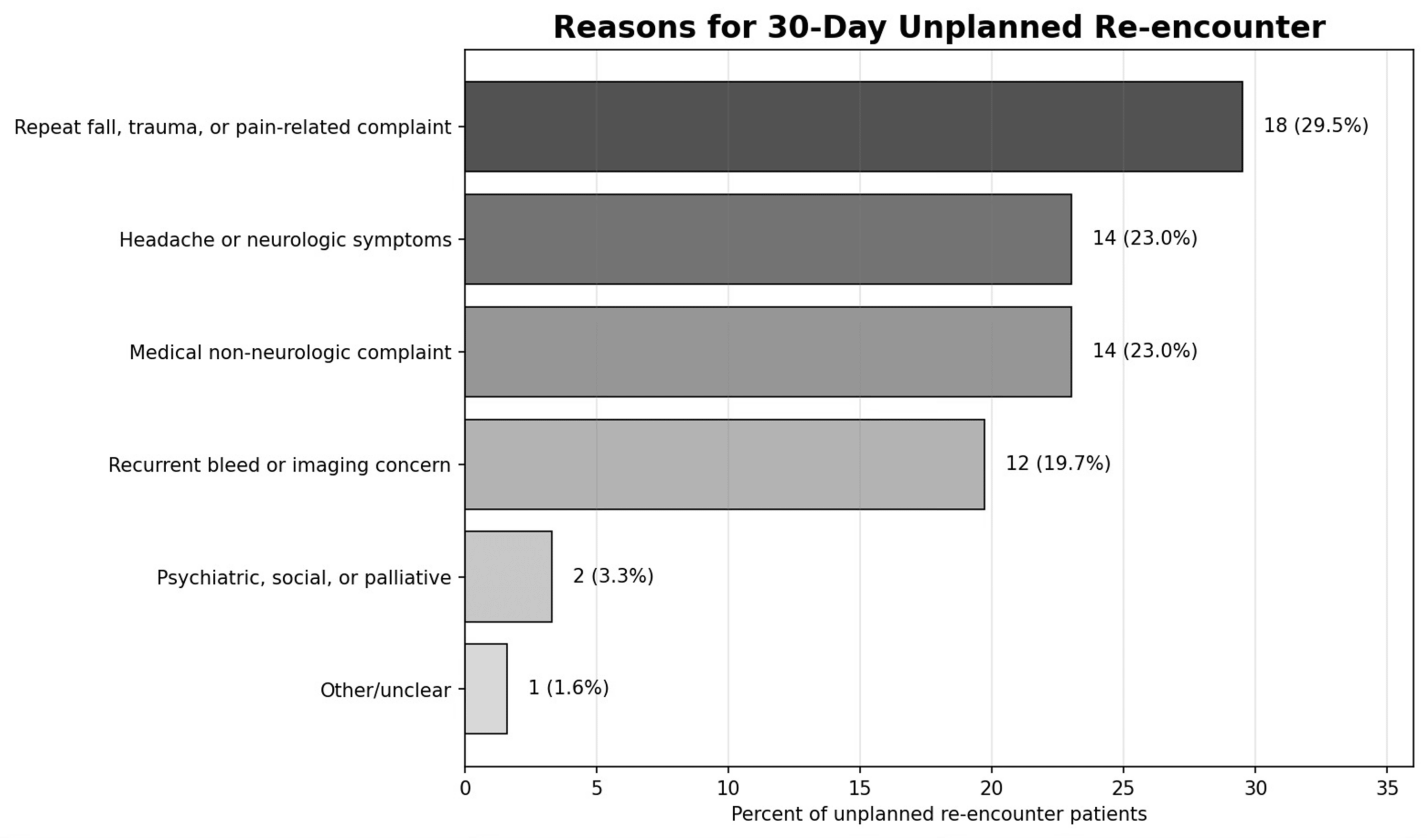
Causes of 30-day unplanned re-encounters This chart shows the reasons for a 30-day unplanned re-encounter. Re-encounters were clinically heterogeneous, with no single category accounting for the majority of return visits.

Among the 61 patients with an unplanned re-encounter, the most common primary reason was repeat fall, trauma, or pain-related complaint (n = 18; 29.5%), followed by medical non-neurologic complaint (n = 14; 23.0%), headache or neurologic symptoms (n = 14; 23.0%), recurrent bleed or imaging concern (n = 12; 19.7%), psychiatric, social, or palliative presentation (n = 2; 3.3%), and other or unclear (n = 1; 1.6%) as described in Figure 2.

## Discussion

This was a retrospective cohort study aimed at describing the baseline medical utilization of minimal head bleed patients managed peripherally via a telemedical protocol within a community trauma system. While telemedical protocols offer an opportunity to reduce unnecessary transfers and improve access to high-quality trauma care, this study aimed to describe the post-discharge management of these peripherally managed patients and provide insight into future quality improvement avenues.

The observed 10.0% readmission rate is broadly consistent with published benchmarks for traumatic brain injury (TBI). Kelly et al. reported a 30-day non-elective readmission rate of 8.9% among 135,542 hospitalized TBI patients in the 2014 Nationwide Readmissions Database [16]. For mild TBI specifically, Ganti et al. reported a 5.2% ED return rate within 72 hours, while Kreitzer et al. found a 13.6% ED return rate within 7 days among mild TBI patients with intracranial hemorrhage [17,18]. The 10.0% readmission rate in the present cohort, which was restricted to minimal head bleeds managed without transfer, falls within the range reported in the broader TBI literature and may suggest that telemedicine-guided management does not appear to produce excess readmission relative to conventionally managed populations.

Although approximately one-quarter of patients returned for unplanned care within 30 days, re-encounters were not significantly associated with age, sex, ISS, or follow-up category. Re-encounters were clinically heterogeneous, spanning 18 (29.5%) recurrent falls or trauma, 14 (23.0%) non-neurologic medical complaints, 14 (23.0%) headache or neurologic symptoms, 12 (19.7%) recurrent bleed or imaging concerns, and 2 (3.3%) psychiatric or social presentations. A patient returning for headache reassurance, a patient returning after a repeat fall, and a patient returning with pneumonia are all captured under the same binary re-encounter outcome and occurred at relatively similar frequencies, but the underlying mechanisms differ substantially. Thus, mechanism-specific interventions to reduce unplanned re-encounters appear challenging based on this dataset.

The absence of statistically significant associations between age, sex, ISS, and post-discharge healthcare utilization in this cohort is notable. Median ISS was identical among readmitted and non-readmitted patients, and neither age nor sex was significantly associated with either outcome. In broader trauma populations, older age and higher injury severity are consistently associated with readmission [16,19]. However, this cohort was intentionally restricted to patients with minimal traumatic intracranial hemorrhage selected for telemedicine-guided management without transfer. Within this relatively low-acuity and narrowly defined population, traditional trauma severity measures do not appear to capture the factors that drive post-discharge utilization. Within this relatively small, low-acuity cohort, standard demographic and injury-severity variables were not significantly associated with post-discharge healthcare utilization. Whether these variables contribute to risk stratification in broader patient populations remains uncertain and warrants evaluation in larger prospective studies.

In contrast, follow-up complexity appeared more informative. Patients requiring any subspecialty follow-up had higher rates of 30-day readmission, and this association persisted after adjustment for age, sex, and ISS. This finding should not be interpreted as evidence that subspecialty follow-up causes readmission. Because follow-up recommendations were determined at the time of the index encounter, patients referred for subspecialty follow-up likely differed from those not referred in ways that were not fully captured by the available covariates. Furthermore, baseline comorbidities, frailty, functional status, and social determinants of health were not available for adjustment, and residual confounding may partially explain the observed associations. Rather, subspecialty follow-up may reflect provider concern, persistent symptoms, associated injuries, comorbid illness, medication complexity, or functional vulnerability at the time of discharge. In this sense, the need for subspecialty care may represent a practical, readily identifiable marker of clinical complexity not captured by ISS or baseline demographics. If validated in larger prospective cohorts, this characteristic could help identify patients for future investigation of targeted transitional-care strategies aimed at reducing post-discharge healthcare utilization. The distinction between surgical and non-surgical subspecialty follow-up adds an important nuance. Surgical subspecialty follow-up was not significantly associated with readmission. However, within this minimal head-bleed injury cohort, non-surgical subspecialty follow-up, such as cardiology or neurology, demonstrated the strongest association with readmission. This again suggests that readmission risk in this cohort may not be driven primarily by procedural injury burden or surgical surveillance needs, but rather an overall global medical complexity. This interpretation is consistent with the American College of Surgeons Best Practices guidelines, which emphasize that patients with mild TBI and prolonged physical, cognitive, or psychological symptoms require multidisciplinary follow-up and rehabilitation referral [20]. Telemedicine-based transitional care models have demonstrated reduced 30-day readmission rates compared with usual care in other populations, and similar approaches may be applicable to this patient group [21]. These interventions may be especially relevant for patients with persistent neurologic symptoms, cardiopulmonary comorbidity, functional limitations, or recurrent fall risk. Patients requiring non-surgical subspecialty follow-up may represent an appropriate population for future studies evaluating structured discharge planning, early telephone outreach, medication reconciliation, fall-risk assessment, symptom surveillance, or coordination between trauma services and outpatient specialists.

Several limitations should be considered when interpreting these findings. First, scheduled follow-up appointments were not standardized and likely reflect provider judgment, patient symptoms, comorbidities, injury pattern, and unmeasured clinical concerns, and introduce several opportunities for bias inherent to many retrospective designs. Next, the dataset represented early outcomes following the implementation of the telemedicine protocol, and the size of the cohort was relatively small, with only 241 total patients. Several of the follow-up categories, such as trauma clinic, were too infrequent, which prohibited a robust statistical analysis of these subcategories. Sensitivity analysis in the form of Firth regression was implemented to improve the validity of these findings, but future analysis on more robust datasets is still warranted. Additionally, because patients were selected for management under a predefined institutional telemedicine protocol, the study cohort represents a carefully selected, lower-acuity population with traumatic intracranial hemorrhage. Consequently, these findings may not be generalizable to patients requiring transfer, neurosurgical intervention, or management at tertiary trauma centers. The dataset also does not include several potentially important predictors, including baseline functional status, frailty, social support, socioeconomic status, and insurance. These are critical factors that may influence the need for post-discharge medical care and should be a focus of future studies. Finally, this study was performed within a single regional trauma system, which may limit generalizability to other telemedicine networks or trauma systems with different referral patterns and outpatient resources.

## Conclusions

Among 241 adults managed through a telemedicine-guided minimal head-bleed protocol, 24/241 (10.0%) experienced 30-day readmission, and 61/241 (25.3%) experienced an unplanned 30-day re-encounter. These rates were comparable to previously reported outcomes in conventionally managed mild traumatic brain injury populations. Age, sex, and injury severity score were not associated with either outcome.

Subspecialty follow-up was associated with increased odds of 30-day readmission after adjustment for age, sex, and injury severity score, with the strongest association observed among patients requiring non-surgical subspecialty care. These findings suggest that subspecialty follow-up may serve as a practical marker of post-discharge clinical complexity after telemedicine-guided management. Larger prospective studies are needed to validate these findings and inform targeted follow-up strategies.
